# Supplementary material for: Stepwise assembly and release of Tc toxins from Yersinia entomophaga
Source: Nat Microbiol. 2024 Feb 5;9(2):405–20. doi: 10.1038/s41564-024-01611-2 (PMC10847046; doi:10.1038/s41564-024-01611-2)
Supplement: Supplementary file 1 — Supplementary Fig. 1–4 and Tables 1–3. [file 41564_2024_1611_MOESM1_ESM.pdf]

nature microbiology

Article

https://doi.org/10.1038/s41564-024-01611-2

Stepwise assembly and release of Tc toxins from *Yersinia entomophaga*

In the format provided by the authors and unedited

Supplementary Information

Supplementary Figures 1 – 4
Supplementary Tables 1 – 3

a

Protein name	Accession number	Exclusive unique peptide counts
Toxin subunit YenA2	A0A3S6EXX9	126
Toxin subunit YenA1	A0A3S6EWV3	81
Toxin subunit YenB	A0A3S6EX30	77
Toxin subunit YenC1	A0A3S6EXC9	62
Toxin subunit YenC2	A0A3S6EWV9	55
Chitinase 1	A0A3S6EWX1	53
Chitinase 2	A0A3S6EWW7	51
RHS2	A0A2D0TC51	22
sfGFP	A0A059PIQ0	17
Peptidase M66/StcE	A0A3S6EYX4	10

b

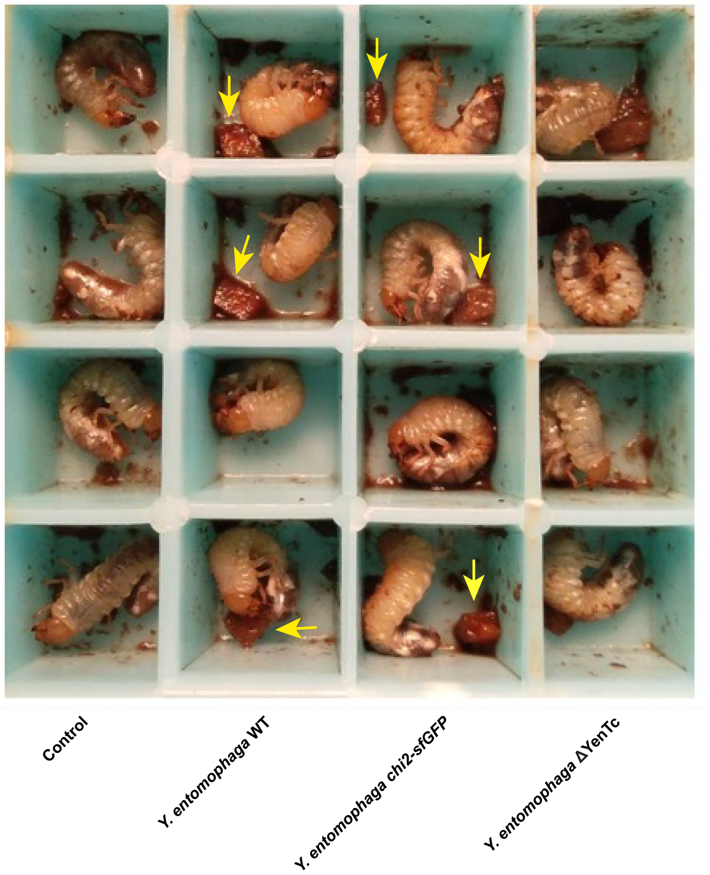

Supplementary Figure 1: Mass Spectrometry and intoxication assays of *Costelytra giveni* indicate that YenTc-sfGFP is active

a: Mass spectrometry analysis of a YenTc purification from *Y. entomophaga chi2-sfGFP* culture identified all YenTc components as well as sfGFP. Note that it also shows the expression of M66/StcE.

b: *Costelytra giveni* larvae were fed with carrot food cubes (yellow arrows) with culture filtrates of different *Y. entomophaga* strains or without filtrate (control). The photo was taken three days after feeding. Larvae fed with carrot without filtrate or with ΔYenTc culture filtrate consumed the entire carrot food cube and were of a healthy grey appearance. Larvae fed with carrot with WT or *chi2-sfGFP* culture filtrate showed a cream coloration and reduced feeding activity.

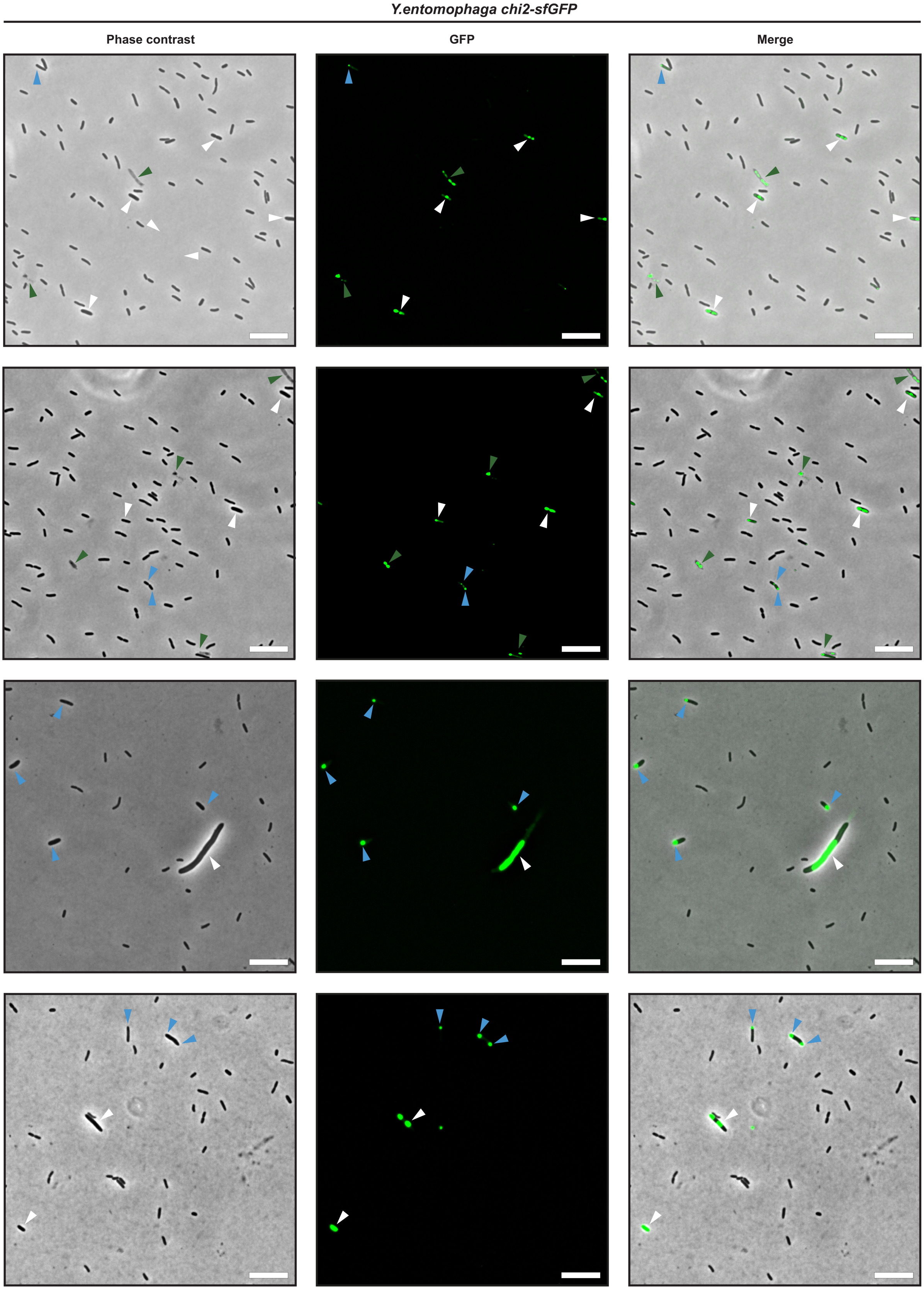

Supplementary Figure 2: More fLM images of *Y. entomophaga chi2-sfGFP* show different morphological categories of Chi2-sfGFP expressing cells

fLM of *Y. entomophaga chi2-sfGFP* grown for 24 h revealed the expression of Chi2-sfGFP only in a subpopulation of cells. Three different morphological classes were detected: Foci at the cell poles (blue arrowheads), distributed signal in somewhat elongated and thicker cells (white arrowheads), and lysing cells (loss of phase contrast, green arrowheads). The experiment was repeated three times for the timepoint 16 h, and four times for the timepoint 24 h with $n_{total,\ 16h}$ = 4810 and $n_{total,\ 24h}$ = 7317 (n > 1000 cells for each experiment). Bar: 10 µm.

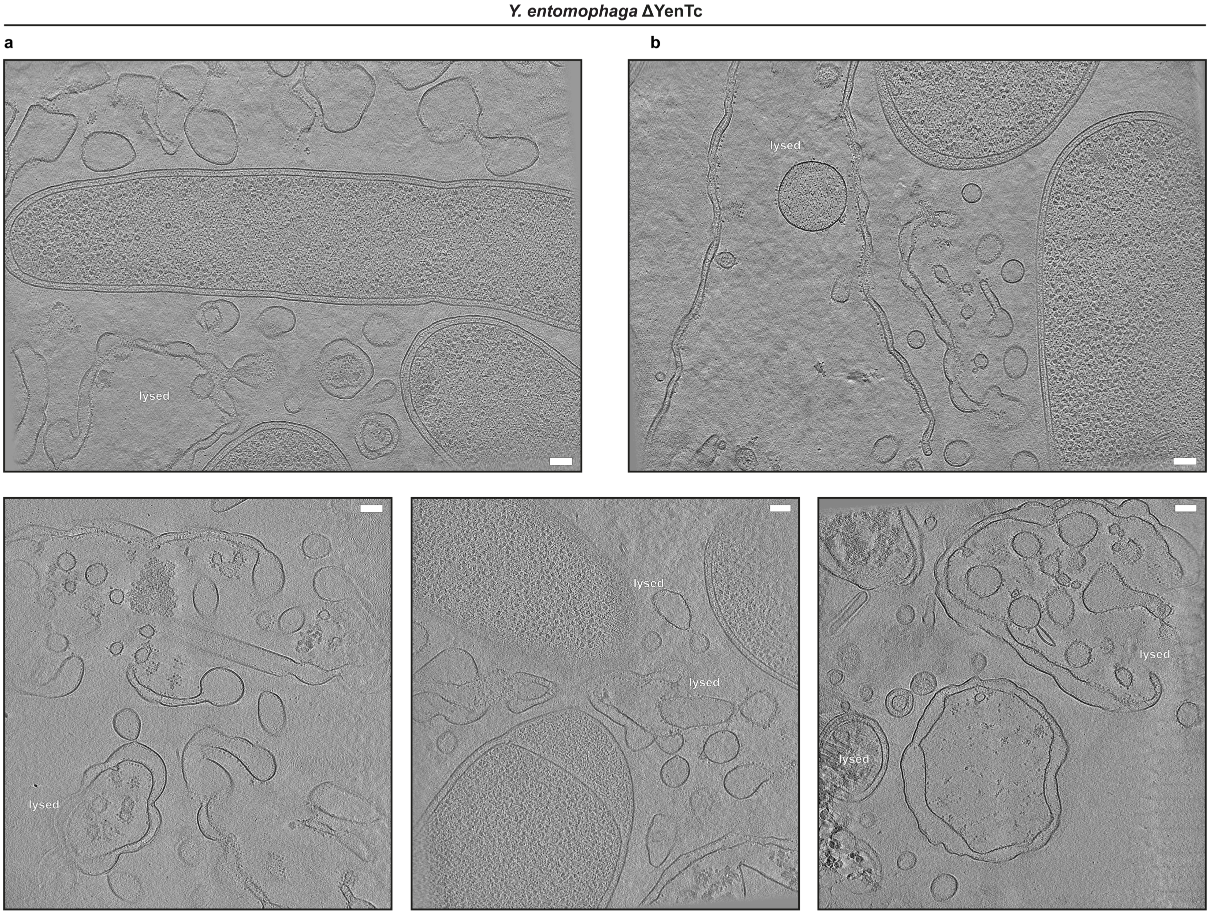

Supplementary Figure 3: Lysed cells of *Y. entomophaga* ΔYenTc do not reveal any YenTc-like densities

Slices through cryo-tomograms of *Y. entomophaga* ΔYenTc cells showed no apparent YenTc densities inside intact or lysed cells (n_{lysed} = 33). Slice thickness: 18.04 nm. Bar: 100 nm.

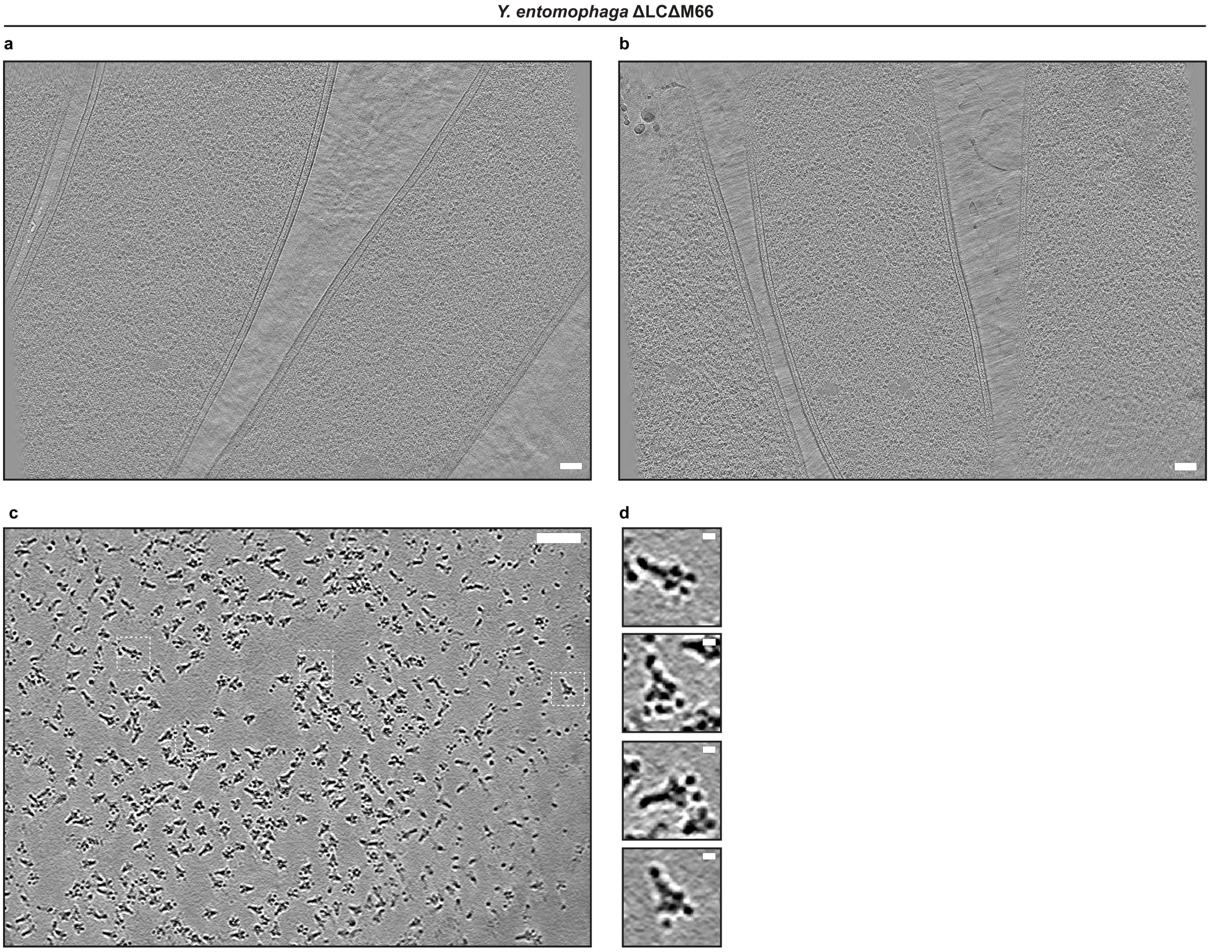

Supplementary Figure 4. ***Y. entomophaga*** **ΔLCΔM66 confirms identity of filaments as M66/StcE and results in holotoxin formation**

a/b: Cryo-tomograms of *Y. entomophaga* ΔLCΔM66 revealed the abolishment of distinct filament bundles in the cytoplasm of the cells (n = 103), confirming the identity of the cytoplasmic filaments as M66. Slice thickness: 18.04 nm. Bar: 100 nm.

c: Slice through cryo-tomograms of a purification of YenTc from *Y. entomophaga* ΔLCΔM66 showed that YenTc can assemble into holotoxins in this mutant. Slice thickness: 18.04 nm. Bar: 100 nm.

d: Close-up views of the boxed YenTc particles from c. Slice thickness: 18.04 nm. Bar: 10 nm.

Supplementary Tables

Supplementary Table 1. Differential expression analysis of ΔLC cells grown at 37 °C compared to 25 °C. FC: fold change, FDR: false discovery rate, Conf.: confidence.

Protein	Accession number	Log_2 FC	FDR	Conf. low	Conf. high
YenA1	A0A3S6EWV3	-7.2	3.49E-5	-9.28	-5.12
YenA2	A0A3S6EXX9	-6.83	2.51E-4	-9.50	-4.16
YenB	A0A3S6EX30	-5.09	7.76E-8	-5.67	-4.51
YenC1	A0A3S6EXC9	-3.17	6.93E-7	-3.69	-2.65
YenC2	A0A3S6EWV9	-6.06	1.08E-5	-7.52	-4.59
Chi1	A0A3S6EWX1	-7.13	1.74E-7	-7.79	-6.48
Chi2	A0A3S6EWW7	-8.25	1.02E-7	-9.25	-7.25
Cbp	A0A3S6EXR6	-6.41	9.97E-9	-6.91	-5.91
Chi3	A0A3S6F1Q8	-3.99	4.38E-7	-4.60	-3.37
Pil36	A0A3S6F569	-3.75	6.45E-5	-4.94	-2.56
PirA	A0A3S6F007	-4.08	1.94E-6	-4.71	-3.44
NucA	A0A3S6F4M5	-5.13	1.3E-4	-6.67	-3.58
M66/StcE	A0A3S6EYX4	-7.05	1.16E-4	-9.51	-4.6
Tlh	A0A3S6F052	-3.22	4.07E-3	-4.93	-1.51
OmpR/PhoB	A0A3S6F5G2	-3.39	7.34E-5	-4.49	-2.29

Supplementary Table 2. Differential expression analysis of WT cells grown at 37 °C compared to 25 °C. FC: fold change, FDR: false discovery rate, Conf.: confidence.

Protein	Accession number	Log_2 FC	FDR	Conf. low	Conf. high
YenA1	A0A3S6EWV3	-6.91	5.09E-5	-8.99	-4.83
YenA2	A0A3S6EXX9	-5.81	7.34E-4	-8.48	-3.14
YenB	A0A3S6EX30	-5.64	5.49E-8	-6.21	-5.06
YenC1	A0A3S6EXC9	-3.53	4.07E-7	-4.05	-3.01
YenC2	A0A3S6EWV9	-4.67	6.53E-5	-6.14	-3.21
Chi1	A0A3S6EWX1	-5.67	9.16E-8	-6.33	-5.02
Chi2	A0A3S6EWW7	-6.83	4.07E-7	-7.84	-5.83
Cbp	A0A3S6EXR6	-4.95	5.49E-8	-5.45	-4.45
Chi3	A0A3S6F1Q8	-2.51	1.20E-5	-3.13	-1.9
Pil36	A0A3S6F569	-3.07	2.47E-4	-4.27	-1.88
PirA	A0A3S6F007	-5.37	2.84E-7	-6	-4.74
NucA	A0A3S6F4M5	-7.05	1.02E-5	-8.6	-5.51
M66/StcE	A0A3S6EYX4	-6.57	1.94E-4	-9.02	-4.12
OmpR/PhoB	A0A3S6F5G2	-3.27	9.62E-5	-4.37	-2.17
Endolysin	A0A3S6F4L4	-2.78	2.86E-2	-5.21	-0.36

Supplementary Table 3. List of primers to generate different *Yersinia entomophaga* mutants

**Mutant**	**Name**	**Sequence (5' to 3')**	**Purpose**
ΔLCΔYenTc	tc_upstream_F	GCTCCTTCGCATTCAT	Confirmation of plasmid insertion in the genome
ΔLCΔYenTc	tc_upstream_R	AGGTAGTGCTATTGTGAC	Confirmation of plasmid insertion in the genome
ΔLCΔYenTc	tc_downstream_F	CTTCCGGCGTAGTGGC	Confirmation of plasmid insertion in the genome
ΔLCΔYenTc	tc_downstream_R	CCCGCCTGCTATAACTGT	Confirmation of plasmid insertion in the genome
ΔLCΔYenTc	yenB_F	GTAAGGTGTTCAATGGTG	Confirmation of the deletion of the Tc toxin gene cluster
ΔLCΔYenTc	yenB_R	AAATCAATTTCTGAAATT AAGAGC	Confirmation of the deletion of the Tc toxin gene cluster
ΔLCΔYenTc	yentc_f1_sal1	ATATATGTCGACAGGCAT AATATGTACCTG	Generation of the plasmid for deletion of the gene cluster
ΔLCΔYenTc	yentc_r1_xho1	ATATATCTCGAGGCGGTT TTCTTTTTTAATCG	Generation of the plasmid for deletion of the gene cluster
ΔLCΔYenTc	yentc_f2	AACGTTGCGCGCTGGTTT CAGACTCCATTTTTTAAA TTA	Generation of the plasmid for deletion of the gene cluster
ΔLCΔYenTc	yentc_f2	TAATTTAAAAAATGGAGT CTGAAACCAGCGCGCAAC GTT	Generation of the plasmid for deletion of the gene
ΔLCΔM66	YenM66_XmaI_F	ATATATCCCGGGATCTTTG TATTGTAATTTTGAGCATC ACAC	Generation of the plasmid for deletion of the gene
ΔLCΔM66	YenM66_F1	AGATGATAATACGCAAGTT AATATCTGTGGACAGAGTT TGATGCCATACTG	Generation of the plasmid for deletion of the gene
ΔLCΔM66	YenM66_R1	CAGTATGGCATCAAACTCT GTCCACAGATATTAACTTG CGTATTATCATCT	Generation of the plasmid for deletion of the gene

ΔLCΔM66	YenM66_NheI_R	ATATATGCTAGCTTCACTGGACCGACGG	Generation of the plasmid for deletion of the gene
ΔLCΔM66	Yen_M66_upstream_F	TTAATGAAAAGCAGCGG	Confirmation of plasmid insertion in the genome
ΔLCΔM66	Yen_M66_upstream_R	CATTTTTAATACCTTATTTAATTGATGT	Confirmation of plasmid insertion in the genome
ΔLCΔM66	Yen_M66_downstream_F	TCCAACCTGGAATTCA	Confirmation of plasmid insertion in the genome
ΔLCΔM66	Yen_M66_downstream_R	CGTACATCCTTTCTGC	Confirmation of plasmid insertion in the genome
ΔLCΔM66	Yen_Mtp_downstream_F	TCCAACCTGGAATTCA	Confirmation of the deletion of M66 gene
ΔLCΔM66	Yen_Mtp_downstream_R	CGTACATCCTTTCTGC	Confirmation of the deletion of M66 gene
chi2-sfGFP	p443_chi2-sfGFP_A1	TGGGTTAAAAAGGATCGATCCTCTAGAGCGCCGATACCAGTATTGAAT	Generation of the plasmid for the Chi2-sfGFP fusion
chi2-sfGFP	chi2-sfGFP_B1	CTTCGCCTTTACGCATTCCGCCACCTGCCGCAGCCTTGCTCTTAATTTCAG	Generation of the plasmid for the Chi2-sfGFP fusion
chi2-sfGFP	chi2-sfGFP_C1	CTGAAATTAAGAGCAAGGCTGCGGCAGGTGGCGGAATGCGTAAAGGCGAAG	Generation of the plasmid for the Chi2-sfGFP fusion
chi2-sfGFP	chi2-sfGFP_D1	TAACAATTGGCGCTGTAAACGTCATTTGTACAGTTCATCC	Generation of the plasmid for the Chi2-sfGFP fusion
chi2-sfGFP	chi2-sfGFP_E1	GGATGAACTGTACAAATGACGTTTACAGCGCCAATTGTTA	Generation of the plasmid for the Chi2-sfGFP fusion
chi2-sfGFP	p443_chi2-sfGFP_F1	GTGAATTCAAAGGGAGAGCTCAGATATCTCGCTCGCCGTAATC	Generation of the plasmid for the Chi2-sfGFP fusion
