# Supplementary material for: Stepwise assembly and release of Tc toxins from Yersinia entomophaga
Source: Nat Microbiol. 2024 Feb 5;9(2):405–20. doi: 10.1038/s41564-024-01611-2 (PMC10847046; doi:10.1038/s41564-024-01611-2)
Supplement: Supplementary file 2 — Reporting Summary [file 41564_2024_1611_MOESM2_ESM.pdf]

nature portfolio

Corresponding author(s): Pilhofer, Martin

Last updated by author(s): Jan 15, 2024

Reporting Summary

Nature Portfolio wishes to improve the reproducibility of the work that we publish. This form provides structure for consistency and transparency in reporting. For further information on Nature Portfolio policies, see our Editorial Policies and the Editorial Policy Checklist.

Statistics

For all statistical analyses, confirm that the following items are present in the figure legend, table legend, main text, or Methods section.

n/a	Confirmed	
☐	☒	The exact sample size (*n*) for each experimental group/condition, given as a discrete number and unit of measurement
☐	☒	A statement on whether measurements were taken from distinct samples or whether the same sample was measured repeatedly
☐	☒	The statistical test(s) used AND whether they are one- or two-sided<br>*Only common tests should be described solely by name; describe more complex techniques in the Methods section.*
☒	☐	A description of all covariates tested
☒	☐	A description of any assumptions or corrections, such as tests of normality and adjustment for multiple comparisons
☐	☒	A full description of the statistical parameters including central tendency (e.g. means) or other basic estimates (e.g. regression coefficient) AND variation (e.g. standard deviation) or associated estimates of uncertainty (e.g. confidence intervals)
☐	☒	For null hypothesis testing, the test statistic (e.g. *F*, *t*, *r*) with confidence intervals, effect sizes, degrees of freedom and *P* value noted<br>*Give P values as exact values whenever suitable.*
☒	☐	For Bayesian analysis, information on the choice of priors and Markov chain Monte Carlo settings
☒	☐	For hierarchical and complex designs, identification of the appropriate level for tests and full reporting of outcomes
☒	☐	Estimates of effect sizes (e.g. Cohen's *d*, Pearson's *r*), indicating how they were calculated

Our web collection on statistics for biologists contains articles on many of the points above.

Software and code

Policy information about availability of computer code

Data collection	cryoET data collection: Serial EM 4.1.0 beta<br>cryoFIB milling: Zeiss SmartFIB 1.14, Zeiss SmartSEM 5.0<br>cryo light microscopy: ZEN Blue (Carl Zeiss Microscopy) version 3.5<br>light microscopy: Leica Application Suite X (Las X) version 3.7.6.25997<br>mass spectrometry: local laboratory information management system (LIMS)
Data analysis	cryo light microscopy: ZEN Blue (Carl Zeiss Microscopy) version 3.5, ZEN Connect (within ZenBlue, Carl Zeiss Microscopy) version 3.5, Fiji 2.9.0<br>mass spectrometry: Scaffold version 5.2.2 (proteome software), local laboratory information management system (LIMS), FragPipe (version 18.0), MSFragger (version 3.5), Philosopher (version 4.4.0), DIA-NN version 1.8.2.<br>cryoET, subtomogram averaging and template matching: IMOD 4.11, MatlabR2022A (9.12.0.1884302), Dynamo version 1.1.532, UCSF Chimera version 1.14, UCSF ChimeraX version 1.6., Relion 4.0, GCTF version 1.06, IsoNet version 0.2, Fiji 2.9.0, AreTomo version 1.3.4, cryoCARE version 0.2.1, DIFFMAP software package 120330, Situs 3.1 (Colores)<br>segmentation: Dragonfly version 2022.2.0.1367, UCSF ChimeraX version 1.6, ArtiaX version 0.1<br>intoxication assay: Minitab version 16.2<br>quantification: GraphPad Prism version 9.5.1, Microsoft Excel version 16.78

For manuscripts utilizing custom algorithms or software that are central to the research but not yet described in published literature, software must be made available to editors and reviewers. We strongly encourage code deposition in a community repository (e.g. GitHub). See the Nature Portfolio guidelines for submitting code & software for further information.

Data

Policy information about availability of data

All manuscripts must include a data availability statement. This statement should provide the following information, where applicable:

- Accession codes, unique identifiers, or web links for publicly available datasets
- A description of any restrictions on data availability
- For clinical datasets or third party data, please ensure that the statement adheres to our policy

Example cryo-tomograms (EMD-18953, EMD-18954, EMD-18955, EMD-18957, EMD-18958, EMD-18960, EMD-18961, EMD-18962, EMD-19370 – EMD-19381) and subtomogram averages (EMD-18970 - EMD-18972) are uploaded to EMDB. All relevant proteomic data are deposited to the ProteomeXchange Consortium via the PRIDE (http://www.ebi.ac.uk/pride) partner repository with the data set identifier PXD048008.

Other datasets used in this study from the UniProt database and AlphaFold protein structure database: A0A3S6EXX9, A0A3S6EWV3, A0A3S6EX30, A0A3S6EXC9, A0A3S6EWV9, A0A3S6EWX1, A0A3S6EWW7, A0A3S6EXR6, A0A3S6F1Q8, A0A3S6F569, A0A3S6F007, A0A3S6F4M5, A0A3S6F5G2, A0A3S6F4L4, A0A3S6EYX4.

Research involving human participants, their data, or biological material

Policy information about studies with human participants or human data. See also policy information about sex, gender (identity/presentation), and sexual orientation and race, ethnicity and racism.

Reporting on sex and gender	N/A
Reporting on race, ethnicity, or other socially relevant groupings	N/A
Population characteristics	N/A
Recruitment	N/A
Ethics oversight	N/A

Note that full information on the approval of the study protocol must also be provided in the manuscript.

Field-specific reporting

Please select the one below that is the best fit for your research. If you are not sure, read the appropriate sections before making your selection.

☒ Life sciences ☐ Behavioural & social sciences ☐ Ecological, evolutionary & environmental sciences

For a reference copy of the document with all sections, see nature.com/documents/nr-reporting-summary-flat.pdf

Life sciences study design

All studies must disclose on these points even when the disclosure is negative.

Sample size

subtomogram averaging:
81 particles found in 11 high-quality tomograms were used for initial in situ subtomogram average of purified YenTc. Final particle number after cross-correlation cleaning: 432.
739 particles found in 3 high-quality tomograms were used for initial subtomogram average of YenTc-Chi2-sfGFP. Final particle number after CC cleaning: 412.
35,332 particles found in 42 high-quality tomograms were used for subtomogram average of the filament particles.

template matching:
Particles (n = 1555 for WT, n = 1133 for chi2-sfGFP) were identified via template matching with a CC cutoff value of 0.2 and 0.17. After visual inspection, n = 304 for WT, n = 567 for chi2-sfGFP were assigned as true positive. Particles (n = 304 for WT, n = 567 for chi2-sfGFP) were used for in situ subtomogram averaging.

cell type quantification via cryoET:
All high-quality tomograms were used for quantification of cells and cell types. Sample size for each dataset is stated in the figure, figure legend (n) or in the text.

light microscopy quantification
All high-quality light-microscopy images were used for quantification of cells and cell types. Sample size for each dataset is stated in the figure or figure legend (n).

intoxication assay: 12 larvae per treatment were used and three independent bioassays were undertaken.

	For all other experiments, no sample size determination was performed.
Data exclusions	Particles which were not fully inside the field of view were excluded. YenTc or YenTc-Chi2-sfGFP seen in tomograms of bad quality were excluded. Overlapping filament structures and filaments seen in tomograms of bad quality were excluded.<br>Template matching: particles which were visually not identified as YenTc like density, such as ice chunks, membranes etc., were assigned as false positive and were excluded.<br>For quantification in cryoET data, only cells with more than half of its size in the field of view were taken into account.
Replication	Replication of cryoET findings was not attempted as it is not applicable.<br>Replications of light microscopy findings were successful at all attempts. All light microscopy findings were replicated at least 3 times.<br>Replications of the intoxication assay were successful at all 3 attempts.<br>All western blots and SDS-PAGEs were replicated at least 3 times with similar results.
Randomization	Extracted particles (for subtomogram averaging) were randomly assigned to two separate groups to calculate half-maps and goldstandard FSC.<br>Light microscopy data was acquired at random locations.<br>For other experiments, no randomization was performed.
Blinding	Particles identified via template matching were blindly distinguished (without knowledge of their cellular localization) between true positive (YenTc like density) and false positive particles (e.g. membrane, ice chunks).<br>For other experiments, blinding was not attempted as it was not feasible.

Reporting for specific materials, systems and methods

We require information from authors about some types of materials, experimental systems and methods used in many studies. Here, indicate whether each material, system or method listed is relevant to your study. If you are not sure if a list item applies to your research, read the appropriate section before selecting a response.

Materials & experimental systems

n/a	Involved in the study
☐	☒ Antibodies
☒	☐ Eukaryotic cell lines
☒	☐ Palaeontology and archaeology
☐	☒ Animals and other organisms
☒	☐ Clinical data
☒	☐ Dual use research of concern
☒	☐ Plants

Methods

n/a	Involved in the study
☒	☐ ChIP-seq
☒	☐ Flow cytometry
☒	☐ MRI-based neuroimaging

Antibodies

Antibodies used	Membranes were incubated with 1 ug ml-1 polyclonal rabbit anti-YenA1 antibody (GenScript), 1:7,500 anti-GFP antibody (ab183734, Abcam), 1:1,000 anti-RecA (ab63797, Abcam) and 1:5000 horseradish peroxidase-conjugated secondary goat anti-rabbit IgG (31460, Invitrogen).
Validation	Validation of antibodies was done by manufacturer (SDS-PAGE of Antigen and Western Blot, Elisa) as well as with western blotting against Yersinia entomophaga wild-type, chi2-sfGFP, lysis cassette deficient and lysis casette deficient/YenTc deletion mutant.

Animals and other research organisms

Policy information about studies involving animals; ARRIVE guidelines recommended for reporting animal research, and Sex and Gender in Research

Laboratory animals	Did not involve laboratory animals.
Wild animals	Costelytra giveni larvae. Field-collected 3rd instar insects were maintained until required in soil stored at 4C.<br>Insects were killed by freezing for 48 hours prior to autoclaving.
Reporting on sex	n/a
Field-collected samples	Did not involve samples collected in the field.
Ethics oversight	No ethics approval was required.

Note that full information on the approval of the study protocol must also be provided in the manuscript.
