# Supplementary figures and images for: Stepwise assembly and release of Tc toxins from Yersinia entomophaga
Source: Nat Microbiol. 2024 Feb 5;9(2):405–20. doi: 10.1038/s41564-024-01611-2 (PMC10847046; doi:10.1038/s41564-024-01611-2)

Unprocessed western blot of Fig. 4 c

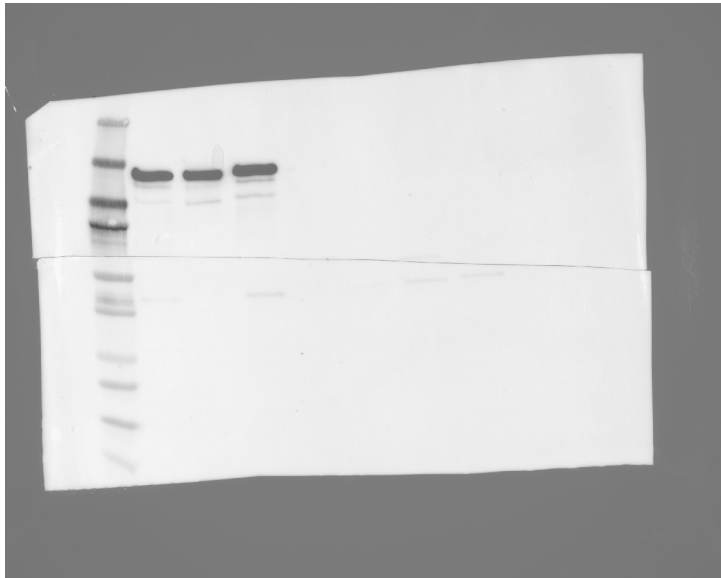

Supplement: Supplementary file 7 — Unprocessed western blot. [file 41564_2024_1611_MOESM7_ESM.pdf]

Unprocessed western blot of Extended Data Fig. 1b

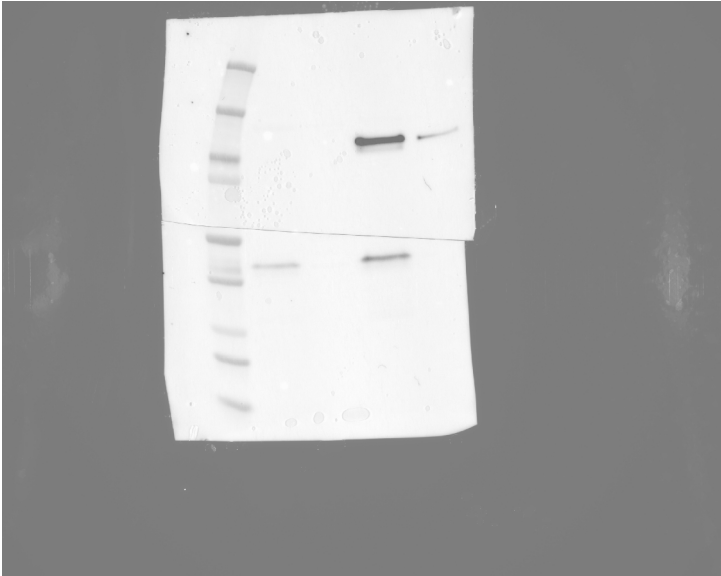

Supplement: Supplementary file 9 — Unprocessed western blot. [file 41564_2024_1611_MOESM9_ESM.pdf]

Unprocessed western blot of Extended Data Fig. 7a

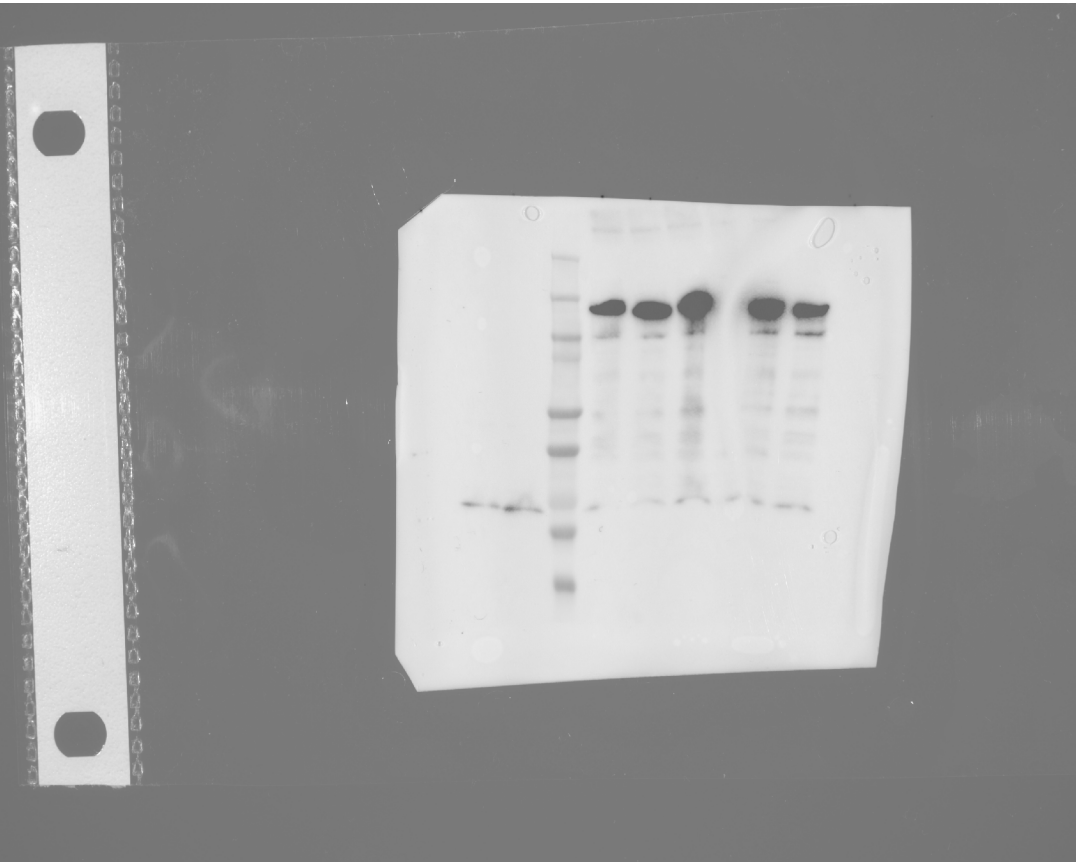

Supplement: Supplementary file 10 — Unprocessed western blot. [file 41564_2024_1611_MOESM10_ESM.pdf]

Unprocessed SDS-PAGE gel of Extended Data Fig. 8a

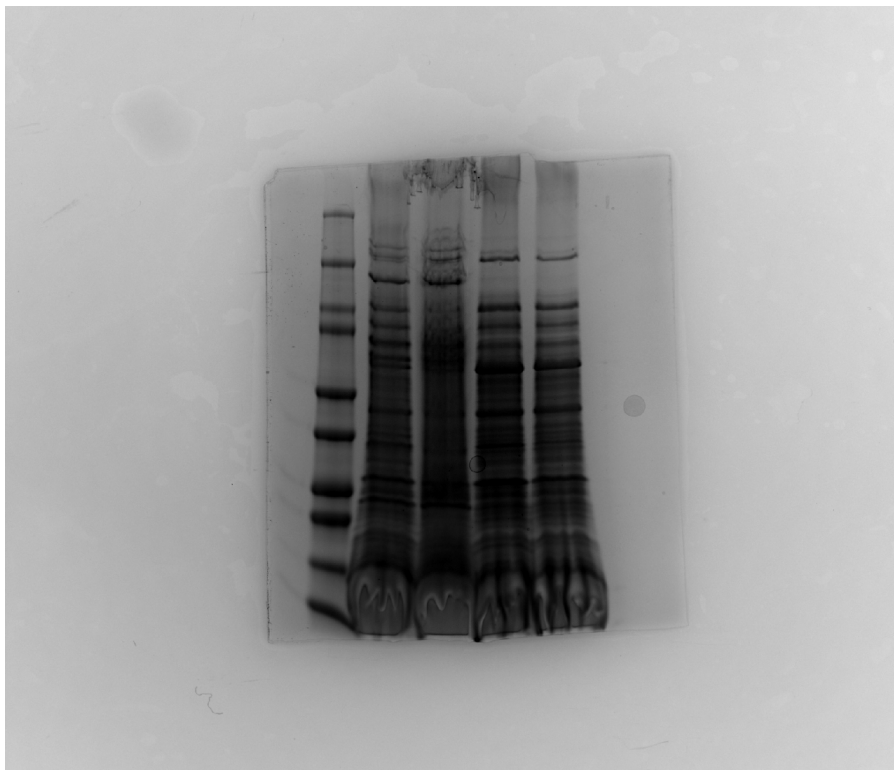

Supplement: Supplementary file 11 — Unprocessed gel. [file 41564_2024_1611_MOESM11_ESM.pdf]

Unprocessed western blot of Extended Data Fig. 8b

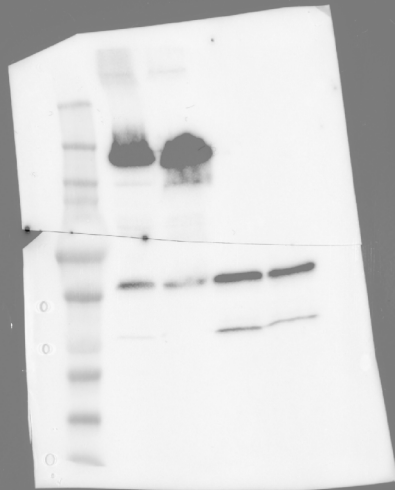

Supplement: Supplementary file 12 — Unprocessed western blot. [file 41564_2024_1611_MOESM12_ESM.pdf]
